# P-428. Impact of Diagnostic Stewardship Interventions on Ventilator-Associated Event Rates

**DOI:** 10.1093/ofid/ofae631.629

**Published:** 2025-01-29

**Authors:** Zachary S Garcia, Jonathan Troost, Owen Albin

**Affiliations:** University of Michigan, Ann Arbor, Michigan; University of Michigan Medical School, Ann Arbor, Michigan; University of Michigan Medical School, Ann Arbor, Michigan

## Abstract

**Background:**

Ventilator-associated events (VAEs) are CDC/NHSN surveillance definitions for monitoring nosocomial complications from invasive mechanical ventilation. In 2022, we executed the Diagnosis of Ventilator-Associated Pneumonia (DIVA) trial (NCT05176353), a quasi-experimental pilot/feasibility clinical trial of a VAP bundled diagnostic stewardship intervention (VAP-DSI) targeting the ICU respiratory culturing pathway. This VAP-DSI associated with a 20% rate reduction in positive respiratory cultures per 1000 mechanically ventilated patient days. In this *post* hoc analysis, we present a secondary data analysis evaluating the impact of a VAP-DSI on VAE surveillance rates.

**Figure:**
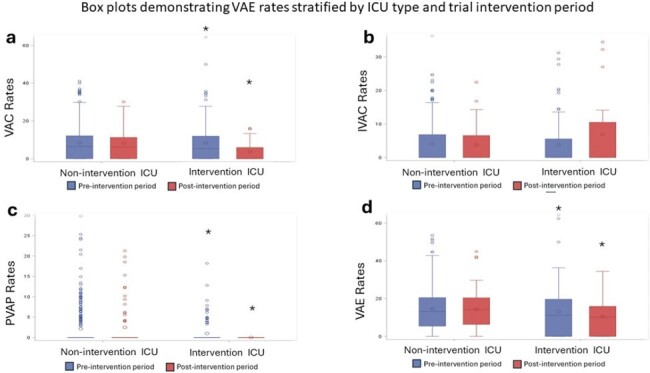

**Methods:**

The DIVA trial operationalized a VAP-DSI using an interruptive clinical decision support tool and modifications to clinical laboratory workflows. Interventions included gatekeeping access to respiratory culture ordering, preferential use of non-bronchoscopic bronchoalveolar lavage for culture collection, and suppression of culture results for samples with minimal alveolar neutrophilia. We compared monthly VAE rates among study ICUs (those that utilized the VAP-DSI) and non-study ICUs over both a 5-year pre-intervention period (2017-2022) and the post-intervention period (2022-2023). Event rates were compared using a Z-test approximation for person-time rates.

**Results:**

Figure 1 demonstrates monthly VAE rates stratified by ICU and pre- vs post-intervention time periods. Study ICUs demonstrated significant decreases in rates of VAEs, VACs, and PVAPs following VAP-DSI implementation. Conversely, non-study ICUs experienced no significant changes in VAE rates.

**Conclusion:**

In this single-center pilot/feasibility clinical trial, diagnostic stewardship interventions targeting the ICU respiratory culturing pathway associated with significant reductions in VACs, PVAPs, and total VAEs. These findings suggest a potential impact of diagnostic stewardship interventions on VAE surveillance rates.

**Disclosures:**

**All Authors**: No reported disclosures

